# 3-[1-(4-Isobutyl­phen­yl)eth­yl]-4-[(*E*)-4-methyl­benzyl­ideneamino]-1*H*-1,2,4-triazole-5(4*H*)-thione

**DOI:** 10.1107/S1600536808036350

**Published:** 2008-11-13

**Authors:** Hoong-Kun Fun, Samuel Robinson Jebas, K. V. Sujith, B. Kalluraya

**Affiliations:** a X-ray Crystallography Unit, School of Physics, Universiti Sains Malaysia, 11800 USM, Penang, Malaysia; b Department of Studies in Chemistry, Mangalore University, Mangalagangotri, Mangalore 574 199, India

## Abstract

In the title compound, C_22_H_26_N_4_S, the dihedral angles formed by the triazole ring with the two benzene rings are 87.51 (3) and 20.98 (3)°. The benzene rings are inclined at 71.88 (2)°. An intra­molecular C—H⋯S hydrogen bond generates an *S*(6) ring motif. The crystal packing is strengthened by inter­molecular N—H⋯S hydrogen bonding and π–π stacking inter­actions between the triazole and benzene rings, with a centroid–centroid distance of 3.6618 (5) Å, together with N⋯N [2.1299 (9)–2.2121 (9) Å] short contacts and C—H⋯π inter­actions. In the crystal packing, mol­ecules are stacked along the *a* axis.

## Related literature

For related literature on componds containing a triazole ring, see: Clemons *et al.* (2004 ▶); Demirbas & Ugurluoglu (2004 ▶); Demirbas *et al.* (2002 ▶); Johnston *et al.* (2002 ▶); Shujuan *et al.* (2004 ▶); For bond-length data, see: Allen *et al.* (1987 ▶). For graph-set analysis of hydrogen bonding, see: Bernstein *et al.* (1995 ▶).
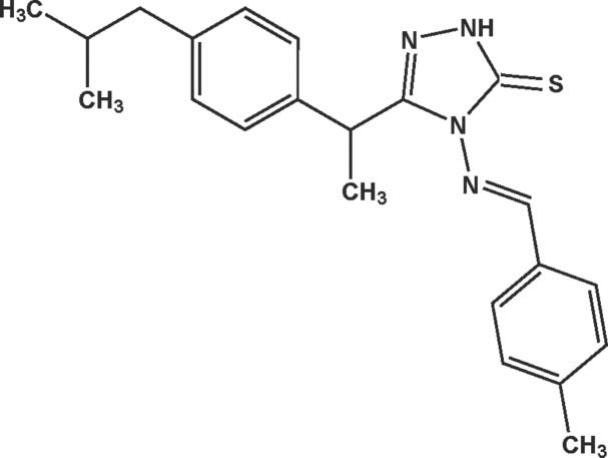


## Experimental

### 

#### Crystal data

C_22_H_26_N_4_S
*M* *_r_* = 378.53
Triclinic, 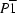

*a* = 7.7614 (2) Å
*b* = 10.7649 (2) Å
*c* = 12.9552 (2) Å
α = 85.900 (1)°
β = 78.575 (1)°
γ = 72.542 (1)°
*V* = 1012.01 (4) Å^3^
*Z* = 2
Mo *K*α radiation
μ = 0.17 mm^−1^
*T* = 100.0 (1) K
0.61 × 0.40 × 0.17 mm

#### Data collection

Bruker SMART APEXII CCD area-detector diffractometer
Absorption correction: multi-scan (*SADABS*; Bruker, 2005 ▶) *T*_min_ = 0.902, *T*_max_ = 0.971
27492 measured reflections
8863 independent reflections
7661 reflections with *I* > 2σ(*I*)
*R*_int_ = 0.021

#### Refinement

*R*[*F* ^2^ > 2σ(*F* ^2^)] = 0.038
*wR*(*F* ^2^) = 0.110
*S* = 1.05
8863 reflections
260 parameters
3 restraints
H atoms treated by a mixture of independent and constrained refinement
Δρ_max_ = 0.62 e Å^−3^
Δρ_min_ = −0.33 e Å^−3^

### 

Data collection: *APEX2* (Bruker, 2005 ▶); cell refinement: *APEX2*; data reduction: *SAINT* (Bruker, 2005 ▶); program(s) used to solve structure: *SHELXTL* (Sheldrick, 2008 ▶); program(s) used to refine structure: *SHELXTL*; molecular graphics: *SHELXTL*; software used to prepare material for publication: *SHELXTL* and *PLATON* (Spek, 2003 ▶).

## Supplementary Material

Crystal structure: contains datablocks global, I. DOI: 10.1107/S1600536808036350/ng2513sup1.cif

Structure factors: contains datablocks I. DOI: 10.1107/S1600536808036350/ng2513Isup2.hkl

Additional supplementary materials:  crystallographic information; 3D view; checkCIF report

## Figures and Tables

**Table 1:** Hydrogen-bond geometry (Å, °)

*D*—H⋯*A*	*D*—H	H⋯*A*	*D*⋯*A*	*D*—H⋯*A*
N2—H1*N*2⋯S1^i^	0.859 (9)	2.411 (9)	3.2619 (7)	171.0 (13)
C10—H10*A*⋯S1	0.93	2.55	3.1834 (8)	126
C12—H12*A*⋯*Cg*2^ii^	0.93	2.70	3.5531 (9)	152
C21—H21*B*⋯*Cg*2^iii^	0.96	2.99	3.8326 (9)	148

Symmetry codes: (i) 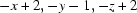; (ii) 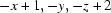; (iii) 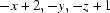. *Cg*2 is the centroid of the C1–C6 ring.

**Fig. 1.**
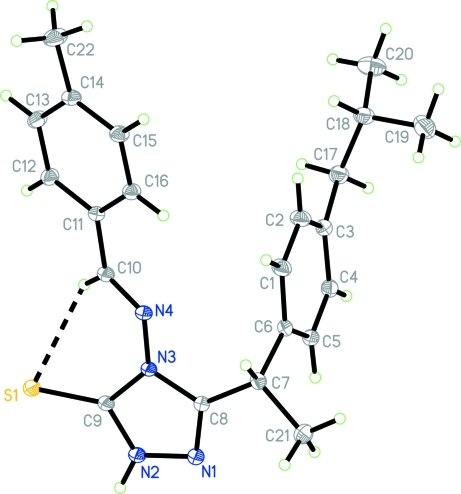
The molecular structure of the title compound, showing 50% probability displacement ellipsoids and the atom numbering scheme.

**Fig. 2.**
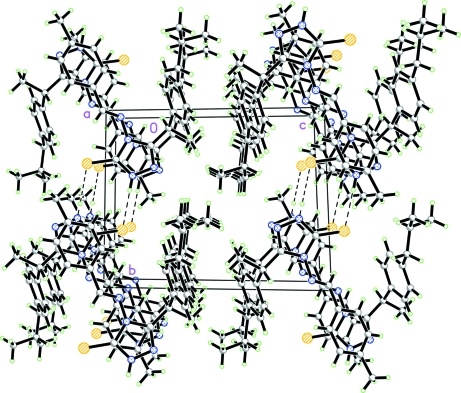
The crystal packing of the title compound,viewed down the a axis.

## supplementary crystallographic information

### Comment

Several compounds containing 1,2,4-triazole rings are well known as drugs. For example, Fluconazole is used as an antimicrobial drug (Shujuan *et al.*, 2004), while Vorozole, Letrozole and Anastrozole are non-steroidal drugs used for the treatment of cancer (Clemons *et al.*, 2004) and Loreclezole is used as an anticonvulsant (Johnston *et al.*, 2002). Some Schiff base derivatives of acetic acid hydrazides containing 1,2,4-triazole-5-one ring have displayed anti-tumor activity against breast cancer, while 2-phenyl ethylideneamino and 2-phenyl ethylamino derivatives of 4-amino-1,2,4-triazol-5-ones have been found to be effective towards lung cell cancer and breast cancer (Demirbas *et al.*, 2004, 2002). Due to the progress that occurs in dealing with the chemistry of substituted 4-amino-1,2,4-triazole-3-thiones and their derivatives as well as their biological activity, we synthesized and here report the crystal structure of 1,2,4-triazole Schiff base.

Bond lengths and angles in (I) (Fig. 1) are found to have normal values (Allen *et al.*, 1987). The two benzene rings are essentially planar with the maximum deviation from planarity being 0.017 (1)Å for atom C6 and 0.013 (1)Å for atom C14 respectively. The dihedral angle formed by the triazole (N1/N2/C9/N3/C8) ring with the two benzene rings (C1—C6; C11—C16) are 87.51 (3)° and 20.98 (3)° respectively. The benzene rings (C1—C6; C11—C16) form dihedral angle of 71.88 (2)°, indicating that they are inclined to each other. An intramolecular C—H···S hydrogen bond generates an S(6) ring motif (Bernstein *et al.*, 1995).

The crystal packing is consolidated by intermolecular N—H···S hydrogen bonding (Table.1). Furthermore the packing is strengthened by π—π stacking interactions involving the triazole (N1/N2/C9/N3/C8) (*Cg*1) ring and the symmetry related (C11—C16) ring (*Cg*3) [*Cg*1···*Cg*3^i^ = 3.6618 (5) Å; symmetry code: (i) 2-*X*,-Y,2-*Z*] together with N···N = 2.1299 (9)–2.2121 (9)Å short contacts and C—H···π interactions. In the crystal packing, the molecules are stacked along the *a* axis (Fig. 2).

### Experimental

The title Schiff-base compound was obtained by refluxing 4-amino-5-[1-(4-isobutylphenyl)ethyl]- 4*H*-1,2,4-triazole-3-thiol (0.01 mol) and 4-methylbenzaldehyde (0.01 mol) in ethanol (50 ml) by adding 3 drops of concentrated Sulfuric acid for 3 h. The solid product obtained was collected by filtration, washed with ethanol and dried. The product obtained was then recrystallized using ethanol. Crystals suitable for X-ray analysis were obtained from acetone–*N*,*N*-dimethylformamide (DMF) (1:3) solution by slow evaporation. (Yield 63%; m.p. 415 K, M.F C_22_H_26_N_4_S)

### Refinement

The amino and methylene H atoms were located in a difference map and refined with restraints of N—H=0.85 (1)Å and C—H=0.96 (1) Å. The remaining H atoms were positioned geometrically [C—H=0.93–0.98Å (aromatic) or 0.96Å (methyl)] and refined using a riding model, with *U*_iso_(H)=1.2U_equ_(aromatic C) and 1.5U_equ_ (methyl C). A rotating group model was used for the methyl group.

### Crystal data

**Table:**

C_22_H_26_N_4_S	*Z* = 2
*M**_r_* = 378.53	*F*_000_ = 404
Triclinic, *P*1	*D*_x_ = 1.242 Mg m^−^^3^
Hall symbol: -P 1	Mo *K*α radiation λ = 0.71073 Å
*a* = 7.7614 (2) Å	Cell parameters from 9961 reflections
*b* = 10.7649 (2) Å	θ = 2.6–26.3º
*c* = 12.9552 (2) Å	µ = 0.17 mm^−^^1^
α = 85.900 (1)º	*T* = 100.0 (1) K
β = 78.575 (1)º	Block, colourless
γ = 72.542 (1)º	0.61 × 0.40 × 0.17 mm
*V* = 1012.01 (4) Å^3^	

### Data collection

**Table:**

Bruker SMART APEXII CCD area-detector diffractometer	8863 independent reflections
Radiation source: fine-focus sealed tube	7661 reflections with *I* > 2σ(*I*)
Monochromator: graphite	*R*_int_ = 0.021
*T* = 100.0(1) K	θ_max_ = 35.0º
φ and ω scans	θ_min_ = 1.6º
Absorption correction: multi-scan(SADABS; Bruker, 2005)	*h* = −12→12
*T*_min_ = 0.902, *T*_max_ = 0.971	*k* = −17→16
27492 measured reflections	*l* = −20→20

### Refinement

**Table:**

Refinement on *F*^2^	Secondary atom site location: difference Fourier map
Least-squares matrix: full	Hydrogen site location: inferred from neighbouring sites
*R*[*F*^2^ > 2σ(*F*^2^)] = 0.038	H atoms treated by a mixture of independent and constrained refinement
*wR*(*F*^2^) = 0.110	*w* = 1/[σ^2^(*F*_o_^2^) + (0.059*P*)^2^ + 0.2347*P*] where *P* = (*F*_o_^2^ + 2*F*_c_^2^)/3
*S* = 1.05	(Δ/σ)_max_ < 0.001
8863 reflections	Δρ_max_ = 0.62 e Å^−^^3^
260 parameters	Δρ_min_ = −0.33 e Å^−^^3^
3 restraints	Extinction correction: none
Primary atom site location: structure-invariant direct methods	

### Special details

**Table:**

Experimental. The data was collected with the Oxford Cyrosystem Cobra low-temperature attachment.
Geometry. All e.s.d.'s (except the e.s.d. in the dihedral angle between two l.s. planes) are estimated using the full covariance matrix. The cell e.s.d.'s are taken into account individually in the estimation of e.s.d.'s in distances, angles and torsion angles; correlations between e.s.d.'s in cell parameters are only used when they are defined by crystal symmetry. An approximate (isotropic) treatment of cell e.s.d.'s is used for estimating e.s.d.'s involving l.s. planes.
Refinement. Refinement of *F*^2^ against ALL reflections. The weighted *R*-factor *wR* and goodness of fit *S* are based on *F*^2^, conventional *R*-factors *R* are based on *F*, with *F* set to zero for negative *F*^2^. The threshold expression of *F*^2^ > σ(*F*^2^) is used only for calculating *R*-factors(gt) *etc*. and is not relevant to the choice of reflections for refinement. *R*-factors based on *F*^2^ are statistically about twice as large as those based on *F*, and *R*- factors based on ALL data will be even larger.

### Fractional atomic coordinates and isotropic or equivalent isotropic displacement parameters (Å^2^)

**Table:**

	*x*	*y*	*z*	*U*_iso_*/*U*_eq_	
S1	0.83244 (3)	−0.312534 (18)	1.075041 (15)	0.01912 (5)	
N1	1.05221 (9)	−0.33177 (6)	0.77577 (5)	0.01633 (12)	
N2	0.98892 (9)	−0.37998 (6)	0.87246 (5)	0.01608 (11)	
N3	0.91368 (8)	−0.17456 (6)	0.89131 (5)	0.01308 (10)	
N4	0.86457 (9)	−0.04642 (6)	0.92483 (5)	0.01403 (11)	
C1	0.77565 (11)	0.10391 (8)	0.71426 (6)	0.01826 (13)	
H1A	0.8435	0.1436	0.7453	0.022*	
C2	0.59831 (11)	0.17417 (8)	0.70084 (7)	0.01935 (14)	
H2A	0.5499	0.2600	0.7231	0.023*	
C3	0.49225 (10)	0.11785 (7)	0.65462 (6)	0.01578 (12)	
C4	0.57282 (11)	−0.00989 (8)	0.61889 (6)	0.01597 (12)	
H4A	0.5068	−0.0488	0.5857	0.019*	
C5	0.75003 (10)	−0.07996 (7)	0.63202 (6)	0.01550 (12)	
H5A	0.8006	−0.1646	0.6072	0.019*	
C6	0.85251 (10)	−0.02490 (7)	0.68188 (5)	0.01447 (12)	
C7	1.03785 (10)	−0.10614 (7)	0.70691 (6)	0.01530 (12)	
H7A	1.0913	−0.0483	0.7367	0.018*	
C8	1.00579 (10)	−0.20638 (7)	0.78940 (5)	0.01400 (12)	
C9	0.90821 (10)	−0.28846 (7)	0.94628 (6)	0.01424 (12)	
C10	0.72862 (10)	−0.01464 (7)	1.00254 (6)	0.01398 (12)	
H10A	0.6706	−0.0763	1.0322	0.017*	
C11	0.66417 (9)	0.11699 (7)	1.04473 (5)	0.01333 (11)	
C12	0.52948 (11)	0.14034 (7)	1.13612 (6)	0.01629 (13)	
H12A	0.4819	0.0738	1.1664	0.020*	
C13	0.46617 (11)	0.26249 (8)	1.18207 (6)	0.01813 (13)	
H13A	0.3773	0.2766	1.2432	0.022*	
C14	0.53423 (10)	0.36416 (7)	1.13763 (6)	0.01632 (13)	
C15	0.66461 (11)	0.34123 (7)	1.04410 (6)	0.01662 (13)	
H15A	0.7078	0.4090	1.0120	0.020*	
C16	0.73062 (10)	0.21915 (7)	0.99839 (6)	0.01555 (12)	
H16A	0.8190	0.2052	0.9370	0.019*	
C17	0.29282 (11)	0.18675 (8)	0.64939 (6)	0.01881 (14)	
C18	0.23940 (11)	0.33496 (8)	0.63355 (7)	0.02000 (14)	
H18A	0.2704	0.3726	0.6916	0.024*	
C19	0.34375 (14)	0.37502 (10)	0.53048 (8)	0.02956 (19)	
H19A	0.3082	0.4683	0.5242	0.044*	
H19B	0.3153	0.3391	0.4724	0.044*	
H19C	0.4735	0.3429	0.5299	0.044*	
C20	0.03200 (13)	0.38811 (10)	0.63867 (9)	0.0304 (2)	
H20A	−0.0020	0.4813	0.6320	0.046*	
H20B	−0.0308	0.3638	0.7049	0.046*	
H20C	−0.0015	0.3526	0.5823	0.046*	
C22	0.47296 (13)	0.49376 (9)	1.19093 (8)	0.02463 (17)	
H22A	0.3411	0.5236	1.2070	0.037*	
H22B	0.5180	0.5557	1.1449	0.037*	
H22C	0.5206	0.4848	1.2549	0.037*	
C21	1.17512 (11)	−0.17176 (9)	0.61034 (6)	0.02119 (15)	
H21A	1.2908	−0.2167	0.6304	0.032*	
H21B	1.1919	−0.1069	0.5577	0.032*	
H21C	1.1287	−0.2329	0.5824	0.032*	
H17A	0.2165 (17)	0.1675 (13)	0.7137 (8)	0.027 (3)*	
H17B	0.2573 (18)	0.1510 (13)	0.5935 (9)	0.027 (3)*	
H1N2	1.0236 (18)	−0.4620 (8)	0.8863 (11)	0.026 (3)*	

### Atomic displacement parameters (Å^2^)

**Table:**

	*U*^11^	*U*^22^	*U*^33^	*U*^12^	*U*^13^	*U*^23^
S1	0.02447 (10)	0.01202 (8)	0.01508 (8)	−0.00159 (7)	0.00310 (6)	0.00220 (6)
N1	0.0200 (3)	0.0127 (3)	0.0138 (2)	−0.0021 (2)	−0.0016 (2)	0.0001 (2)
N2	0.0210 (3)	0.0098 (2)	0.0149 (2)	−0.0019 (2)	−0.0014 (2)	−0.00002 (19)
N3	0.0153 (2)	0.0094 (2)	0.0127 (2)	−0.00191 (19)	−0.00099 (18)	0.00014 (18)
N4	0.0164 (2)	0.0096 (2)	0.0144 (2)	−0.0017 (2)	−0.00228 (19)	−0.00062 (19)
C1	0.0216 (3)	0.0136 (3)	0.0202 (3)	−0.0035 (3)	−0.0077 (3)	−0.0007 (2)
C2	0.0223 (3)	0.0130 (3)	0.0222 (3)	−0.0010 (3)	−0.0081 (3)	−0.0031 (3)
C3	0.0184 (3)	0.0140 (3)	0.0139 (3)	−0.0027 (2)	−0.0038 (2)	−0.0002 (2)
C4	0.0193 (3)	0.0145 (3)	0.0142 (3)	−0.0047 (2)	−0.0037 (2)	−0.0001 (2)
C5	0.0195 (3)	0.0121 (3)	0.0136 (3)	−0.0033 (2)	−0.0022 (2)	−0.0002 (2)
C6	0.0172 (3)	0.0123 (3)	0.0124 (3)	−0.0030 (2)	−0.0022 (2)	0.0017 (2)
C7	0.0158 (3)	0.0144 (3)	0.0141 (3)	−0.0033 (2)	−0.0014 (2)	0.0019 (2)
C8	0.0149 (3)	0.0126 (3)	0.0125 (3)	−0.0016 (2)	−0.0017 (2)	0.0001 (2)
C9	0.0160 (3)	0.0103 (3)	0.0145 (3)	−0.0022 (2)	−0.0013 (2)	0.0009 (2)
C10	0.0149 (3)	0.0109 (3)	0.0152 (3)	−0.0027 (2)	−0.0025 (2)	0.0000 (2)
C11	0.0138 (3)	0.0112 (3)	0.0139 (3)	−0.0021 (2)	−0.0025 (2)	−0.0004 (2)
C12	0.0187 (3)	0.0133 (3)	0.0156 (3)	−0.0047 (2)	0.0001 (2)	−0.0011 (2)
C13	0.0195 (3)	0.0156 (3)	0.0172 (3)	−0.0042 (3)	0.0011 (2)	−0.0034 (2)
C14	0.0168 (3)	0.0122 (3)	0.0189 (3)	−0.0023 (2)	−0.0031 (2)	−0.0027 (2)
C15	0.0179 (3)	0.0122 (3)	0.0191 (3)	−0.0043 (2)	−0.0019 (2)	−0.0007 (2)
C16	0.0161 (3)	0.0128 (3)	0.0165 (3)	−0.0038 (2)	−0.0008 (2)	−0.0007 (2)
C17	0.0179 (3)	0.0180 (3)	0.0196 (3)	−0.0027 (3)	−0.0049 (2)	−0.0013 (3)
C18	0.0183 (3)	0.0173 (3)	0.0218 (3)	−0.0004 (3)	−0.0048 (3)	−0.0015 (3)
C19	0.0284 (4)	0.0265 (4)	0.0291 (4)	−0.0038 (3)	−0.0043 (3)	0.0082 (3)
C20	0.0196 (4)	0.0248 (4)	0.0423 (5)	0.0017 (3)	−0.0076 (3)	−0.0024 (4)
C22	0.0285 (4)	0.0154 (3)	0.0273 (4)	−0.0048 (3)	0.0010 (3)	−0.0070 (3)
C21	0.0191 (3)	0.0231 (4)	0.0175 (3)	−0.0038 (3)	0.0017 (2)	0.0005 (3)

### Geometric parameters (Å, °)

**Table:**

S1—C9	1.6843 (7)	C12—C13	1.3904 (11)
N1—C8	1.3039 (10)	C12—H12A	0.9300
N1—N2	1.3769 (9)	C13—C14	1.3945 (11)
N2—C9	1.3434 (9)	C13—H13A	0.9300
N2—H1N2	0.859 (8)	C14—C15	1.4001 (11)
N3—C9	1.3809 (9)	C14—C22	1.5030 (11)
N3—C8	1.3839 (9)	C15—C16	1.3883 (10)
N3—N4	1.3932 (9)	C15—H15A	0.9300
N4—C10	1.2868 (9)	C16—H16A	0.9300
C1—C6	1.3937 (11)	C17—C18	1.5328 (12)
C1—C2	1.3957 (11)	C17—H17A	0.970 (8)
C1—H1A	0.9300	C17—H17B	0.965 (8)
C2—C3	1.3966 (11)	C18—C19	1.5243 (13)
C2—H2A	0.9300	C18—C20	1.5276 (12)
C3—C4	1.3988 (11)	C18—H18A	0.9800
C3—C17	1.5153 (11)	C19—H19A	0.9600
C4—C5	1.3933 (11)	C19—H19B	0.9600
C4—H4A	0.9300	C19—H19C	0.9600
C5—C6	1.3937 (11)	C20—H20A	0.9600
C5—H5A	0.9300	C20—H20B	0.9600
C6—C7	1.5260 (10)	C20—H20C	0.9600
C7—C8	1.5031 (10)	C22—H22A	0.9600
C7—C21	1.5317 (11)	C22—H22B	0.9600
C7—H7A	0.9800	C22—H22C	0.9600
C10—C11	1.4607 (10)	C21—H21A	0.9600
C10—H10A	0.9300	C21—H21B	0.9600
C11—C12	1.3973 (10)	C21—H21C	0.9600
C11—C16	1.3997 (11)		
			
C8—N1—N2	104.10 (6)	C12—C13—H13A	119.6
C9—N2—N1	113.95 (6)	C14—C13—H13A	119.6
C9—N2—H1N2	123.7 (9)	C13—C14—C15	118.39 (7)
N1—N2—H1N2	120.6 (9)	C13—C14—C22	120.58 (7)
C9—N3—C8	108.26 (6)	C15—C14—C22	121.01 (7)
C9—N3—N4	131.25 (6)	C16—C15—C14	121.17 (7)
C8—N3—N4	119.96 (6)	C16—C15—H15A	119.4
C10—N4—N3	115.74 (6)	C14—C15—H15A	119.4
C6—C1—C2	120.96 (7)	C15—C16—C11	119.99 (7)
C6—C1—H1A	119.5	C15—C16—H16A	120.0
C2—C1—H1A	119.5	C11—C16—H16A	120.0
C1—C2—C3	121.17 (7)	C3—C17—C18	117.04 (7)
C1—C2—H2A	119.4	C3—C17—H17A	108.6 (8)
C3—C2—H2A	119.4	C18—C17—H17A	108.0 (8)
C2—C3—C4	117.51 (7)	C3—C17—H17B	109.9 (8)
C2—C3—C17	122.25 (7)	C18—C17—H17B	107.4 (8)
C4—C3—C17	120.10 (7)	H17A—C17—H17B	105.3 (11)
C5—C4—C3	121.31 (7)	C19—C18—C20	110.91 (8)
C5—C4—H4A	119.3	C19—C18—C17	112.08 (7)
C3—C4—H4A	119.3	C20—C18—C17	109.57 (8)
C4—C5—C6	120.88 (7)	C19—C18—H18A	108.0
C4—C5—H5A	119.6	C20—C18—H18A	108.0
C6—C5—H5A	119.6	C17—C18—H18A	108.0
C1—C6—C5	118.09 (7)	C18—C19—H19A	109.5
C1—C6—C7	120.91 (7)	C18—C19—H19B	109.5
C5—C6—C7	120.86 (7)	H19A—C19—H19B	109.5
C8—C7—C6	108.52 (6)	C18—C19—H19C	109.5
C8—C7—C21	110.40 (6)	H19A—C19—H19C	109.5
C6—C7—C21	113.55 (6)	H19B—C19—H19C	109.5
C8—C7—H7A	108.1	C18—C20—H20A	109.5
C6—C7—H7A	108.1	C18—C20—H20B	109.5
C21—C7—H7A	108.1	H20A—C20—H20B	109.5
N1—C8—N3	110.74 (6)	C18—C20—H20C	109.5
N1—C8—C7	126.21 (7)	H20A—C20—H20C	109.5
N3—C8—C7	123.00 (6)	H20B—C20—H20C	109.5
N2—C9—N3	102.85 (6)	C14—C22—H22A	109.5
N2—C9—S1	127.09 (6)	C14—C22—H22B	109.5
N3—C9—S1	129.97 (6)	H22A—C22—H22B	109.5
N4—C10—C11	120.87 (7)	C14—C22—H22C	109.5
N4—C10—H10A	119.6	H22A—C22—H22C	109.5
C11—C10—H10A	119.6	H22B—C22—H22C	109.5
C12—C11—C16	119.12 (7)	C7—C21—H21A	109.5
C12—C11—C10	117.53 (7)	C7—C21—H21B	109.5
C16—C11—C10	123.35 (6)	H21A—C21—H21B	109.5
C13—C12—C11	120.40 (7)	C7—C21—H21C	109.5
C13—C12—H12A	119.8	H21A—C21—H21C	109.5
C11—C12—H12A	119.8	H21B—C21—H21C	109.5
C12—C13—C14	120.87 (7)		
			
C8—N1—N2—C9	−1.39 (9)	C6—C7—C8—N3	−66.34 (9)
C9—N3—N4—C10	−33.22 (11)	C21—C7—C8—N3	168.62 (7)
C8—N3—N4—C10	156.15 (7)	N1—N2—C9—N3	2.75 (9)
C6—C1—C2—C3	0.00 (13)	N1—N2—C9—S1	−174.08 (6)
C1—C2—C3—C4	−2.26 (12)	C8—N3—C9—N2	−2.97 (8)
C1—C2—C3—C17	173.47 (8)	N4—N3—C9—N2	−174.43 (7)
C2—C3—C4—C5	2.09 (11)	C8—N3—C9—S1	173.73 (6)
C17—C3—C4—C5	−173.73 (7)	N4—N3—C9—S1	2.27 (12)
C3—C4—C5—C6	0.34 (11)	N3—N4—C10—C11	−179.96 (6)
C2—C1—C6—C5	2.44 (12)	N4—C10—C11—C12	−173.19 (7)
C2—C1—C6—C7	−173.48 (7)	N4—C10—C11—C16	6.71 (11)
C4—C5—C6—C1	−2.61 (11)	C16—C11—C12—C13	−1.82 (11)
C4—C5—C6—C7	173.32 (7)	C10—C11—C12—C13	178.09 (7)
C1—C6—C7—C8	108.52 (8)	C11—C12—C13—C14	0.56 (12)
C5—C6—C7—C8	−67.29 (9)	C12—C13—C14—C15	1.58 (12)
C1—C6—C7—C21	−128.32 (8)	C12—C13—C14—C22	−176.58 (8)
C5—C6—C7—C21	55.87 (9)	C13—C14—C15—C16	−2.50 (12)
N2—N1—C8—N3	−0.65 (8)	C22—C14—C15—C16	175.66 (8)
N2—N1—C8—C7	−178.19 (7)	C14—C15—C16—C11	1.26 (12)
C9—N3—C8—N1	2.37 (9)	C12—C11—C16—C15	0.92 (11)
N4—N3—C8—N1	174.96 (6)	C10—C11—C16—C15	−178.99 (7)
C9—N3—C8—C7	180.00 (7)	C2—C3—C17—C18	35.20 (11)
N4—N3—C8—C7	−7.40 (10)	C4—C3—C17—C18	−149.19 (7)
C6—C7—C8—N1	110.92 (8)	C3—C17—C18—C19	60.72 (10)
C21—C7—C8—N1	−14.12 (11)	C3—C17—C18—C20	−175.71 (7)

### Hydrogen-bond geometry (Å, °)

**Table:**

*D*—H···*A*	*D*—H	H···*A*	*D*···*A*	*D*—H···*A*
N2—H1N2···S1^i^	0.859 (9)	2.411 (9)	3.2619 (7)	171.0 (13)
C10—H10A···S1	0.93	2.55	3.1834 (8)	126
C12—H12A···Cg2^ii^	0.93	2.70	3.5531 (9)	152
C21—H21B···Cg2^iii^	0.96	2.99	3.8326 (9)	148

Symmetry codes: (i) −*x*+2, −*y*−1, −*z*+2; (ii) −*x*+1, −*y*, −*z*+2; (iii) −*x*+2, −*y*, −*z*+1.
